# Courtship and Reproduction of the Whitetip Reef Shark *Triaenodon obesus* (Carcharhiniformes: Carcharhinidae) in an Ex Situ Environment, with a Description of the Late Embryonic Developmental Stage

**DOI:** 10.3390/ani12233291

**Published:** 2022-11-25

**Authors:** Sérgio Ricardo Santos, Veronica Takatsuka, Shayra P. Bonatelli, Nicole L. L. Amaral, Matheus F. Goés, Rafael F. Valle

**Affiliations:** 1 Instituto Museu Aquário Marinho do Rio de Janeiro—IMAM/AquaRio, Praça Muhammad Ali, Gambôa, Rio de Janeiro 20220-360, Brazil; 2 Laboratório de Biologia e Tecnologia Pesqueira—BioTecPesca, Universidade Federal do Rio de Janeiro (UFRJ), Av. Carlos Chagas Filho, 373—Sala A1-083, Cidade Universitária, Rio de Janeiro 21941-902, Brazil; 3 RADIUS—Centro Especializado em Diagnóstico por Imagem Veterinário, R. José Siqueira, 156—Sala 2, Dom Bosco, Itajaí 88307-310, Brazil

**Keywords:** ultrasound, elasmobranch, gestational, aquarium, conservation

## Abstract

**Simple Summary:**

The reproduction of key reef species is still largely unknown due to difficulties in documenting all elements and steps involved. Sharks are particularly affected by this scarcity of information due to being long-lived species, and witnessing courtship, gestation, and birth is still mostly limited to fortuitous encounters by divers or specimens captured by fishers. Still scarcely described in the literature, our study reports the successful reproduction of *Triaenodon obesus* in an ex situ environment, which offers an opportunity to observe all steps of the reproduction in detail. Furthermore, we offer the first description of the late embryonic developmental stage based on ultrasound imagery.

**Abstract:**

Elasmobranchs represent a group of species under considerable anthropic pressure because of the scale of industrial and artisanal fisheries and the loss of essential areas for nursery and feeding, which are causing substantial population losses around the world. Reproduction in an ex situ environment enables a healthy population to be built and maintained in networks of public aquariums, increasing our knowledge of elasmobranch reproductive biology and offering the opportunity for reintroductions in areas where native populations have been removed. The study reports two successful pregnancies of the whitetip reef shark *Triaenodon obesus*, considered a vulnerable species by the International Union for the Conservation of Nature. Copulation and gestation data are provided, including ultrasound recordings of the late stage of embryo development. Ultrasonography was performed with the GE Logiq and convex transducer and revealed a fetus with defined fins and organogenesis, with definition of eyes, gills, liver, a heart with individualized chambers, partially defined kidneys, and a well-defined spiral intestine. A cartilaginous skeleton forming a posterior acoustic shadow was detailed, as well as a moving fetus with a biparietal diameter of 6.47 cm and a heart rate of 62 Beats Per Minute on spectral Doppler. This is the first successful reproduction of *T. obesus* in an aquarium in Brazil.

## 1. Introduction

Declines in elasmobranch populations related to diverse and continuous anthropic impacts have been widely reported in the ecosystems of several parts of the world [1,2,3,4]. These losses represent only a fraction of the actual damage suffered by populations in these ecosystems, where elasmobranchs represent important components of the food web [5,6,7,8]. Fishery impacts are also underestimated, given the scarcity of data for most marine ecosystems, particularly in tropical waters and off the coasts of developing countries, with numerous unmonitored fishing communities associated with small-scale fisheries [9]. The growing popular appeal of the conservation of marine environments, the public demand to establish the extent of environmental changes, and the worsening of the conservation status of several flagship species of elasmobranchs have promoted the growth of sustainable management plans in the last decades. A compromise between governments and non-governmental organizations (NGOs) was established based on commitments assumed since the Rio 1992 convention [10,11], yet the environmental policies and the species protection efforts implemented in the following decades have still been unable to revert or mitigate the population declines of several species. Special interest has been devoted to the conservation of coral reefs and their inhabitants, considering the fragility of these ecosystems in the face of climate change and a wide variety of human impacts, with the whitetip reef shark *Triaenodon obesus* (Rüppell, 1837) being a known representative.

Despite being a coastal demersal species with a limited dispersal capacity and home range, *T. obesus* is the most widely distributed reef shark species in the world, found from the Red Sea to Micronesia, as well as from the coast of Mexico to Panama in the Eastern Pacific, and its presence is suspected even in the southwestern Atlantic [12,13,14]. This wide distribution, however, does not mean that the species is not threatened. The continuous pressure exerted by human impacts on the ecosystems they inhabit, whether through direct activities such as fishing [15] or through environmental degradation and loss of habitat [16,17], exposes populations of this species to conditions hostile to their survival. The situation is worsening in the context of climate change and its effects on marine ecosystems, within which coral reefs are especially impacted areas [16,18]. As a direct consequence of this situation, the decline seen in the last three generations of whitetip reef shark populations led to the classification of their conservation status as ‘vulnerable’ by the Red List of the International Union for Conservation of Nature (IUCN) [19].

*Triaenodon obesus*, despite its wide distribution, is assessed as vulnerable due to population decline and habitat loss in many areas of its range [20,21], but gaps in knowledge persist. The whitetip reef shark is a placentotrophic viviparous species, with a matrotrophic nutrition of the fetus [22,23]. That is, *T. obesus* is a live-bearing shark with the development of the embryo sustained by nutrients produced by the mother and delivered by a placental connection, a feature only identified within five families of Carcharhiniformes (Carcharhinidae, Sphyrnidae, Hemigaleidae, Leptochariidae, and Triakidae) [22]. Recently, ultrasound studies on elasmobranchs in captivity were published, focusing mainly on determining gestation and general anatomy [24,25,26,27]. However, there is a scarcity of studies focused on the description of the ultrasonographic aspects of fetal organs and their organogenesis in sharks. Since *T. obesus* is considered vulnerable, the detailed monitoring of pregnancy is of paramount importance for the preservation of the species.

While the high cost of field studies hinders the in situ observation of the species, alternative sources are available. The limited home range, average size of adult specimens, and ease of access and exchange among public aquariums have made the species a common sight in public exhibitions around the world [28]. The need for data that support the handling of *T. obesus* under human care and the knowledge about the biology of the species that can be acquired in public aquariums and research centers, especially by monitoring aspects that are difficult to track in its natural environment, such as reproduction, make ex situ studies fundamental in reversing its population decline. The present study describes the reproductive success at the Rio de Janeiro Marine Aquarium (AquaRio) in 2022 with the birth of a male and a female specimen of whitetip reef shark, as well as presenting continuous monitoring of the courtship, gestation, parturition, and postpartum processes. Due to the scarcity of ultrasound data of pregnancy for the species, the objective of the work also included a description of the fetal structures in the final stage of gestation.

## 2. Materials and Methods

The Rio de Janeiro Marine Aquarium’s breeding stock consists of more than 2600 specimens of 306 species of marine and freshwater organisms, with an emphasis on 67 individuals of 11 species of Elasmobranchii. The whitetip reef shark *T. obesus* was originally represented by five specimens (three females, two males) from the coast of Indonesia that were transported to Brazil in August 2017. At the time, all specimens were smaller than 90 cm TL (total length), which identified all individuals as juveniles, considering the size at first maturation reported in the literature (♂ = 104 cm TL, ♀ = 105 cm TL) [29]. After a period of quarantine, the sharks were introduced into the 3.5 million-liter marine exhibition tank, with a maximum depth of 7 m, where they have been kept for the past four years. The oceanic tank is equipped with a mechanical filtration system comprising 20 sand filters, 9 skimmers (Altamar 25c) with ozone injection (O3R and Altamar, 40 g/h), and a degasser (1200 gal/h). Reverse bottom biological filtration is complemented by weekly siphoning. Temperature control is achieved via a heat exchanger. The diet of whitetip reef shark adults is offered daily and includes *Euthynnus alletteratus* (Rafinesque, 1810), *Coryphaena hippurus* Linnaeus, 1758, *Sardinella brasiliensis* (Steindachner, 1879), *Cynoscion* spp., *Anchoa* spp., *Pseudupeneus maculatus* (Bloch, 1793), and *Dorytheutis* spp. Feeding is unrestricted three times a week at four points in the oceanic tank where specimens of *T. obesus* have been conditioned to go.

Two observation strategies were used on our protocol: The first one comprises two programmed daily visits on the entire perimeter of the oceanic tank performed by a veterinarian or an aquarist. The ethogram records any deviation in behavior from the known baseline for each elasmobranch. The second source of observations derives from opportunistic records (i.e., video, photographs, or reports) from environmental educators, a group comprised of a dozen professionals that are continuously present around the oceanic tank for ten hours a day. When isolated, the pregnant female is closely monitored throughout the day by a team of aquarists and veterinarians.

### Ultrasound Examination

The ultrasound examination of the first parturient whitetip reef shark female was performed using a Logiq e model (General Electric^®^, Boston, MA, USA) equipped with a convex transducer ranging in frequency from 3 to 5 MHz. Latex gloves coated with a generous amount of gel were used to protect the transducer from possible damage caused by contact with salt water and shark skin and to promote adequate transmission of the ultrasound wave. The animal was physically restrained using the tonic immobility maneuver and positioned in dorsal decubitus throughout the examination, according to the structure being analyzed and with the patient’s cooperation. A state also known as animal hypnosis, it induces loss of muscle tone and equilibrium while the shark remains unresponsive to major stimulation. It allows safer and less stressful procedures and, unlike chemical anesthetics, allows immediate recovery and minimal disruption to respiration [30,31]. An ultrasound scan of the entire coelomic cavity, the diagnosis of pregnancy, and the detailing of the fetus were performed. Anatomical descriptions follow Crow and Brock [32] and Mylniczenko [23].

## 3. Results

### 3.1. Courtship, Copulation, and Pregnancy

The presence of bite scars on the female’s fins was noticed in the first days of June 2021, and the first courtship with copulation was recorded on 2 June 2021. The event lasted for 3 min 55 s from the moment of the bite until the removal of the clasper and separation of participants (Video S1). The courtship began with the male’s pursuit of the female until the male was anchored by biting the female’s left pectoral fin, starting a spiral descent to the bottom of the tank with slow or no swimming. Still in the water column, the male inserted its right clasper into the female’s cloaca, remaining attached until the end of copulation. The position of the couple varied throughout the process, with the snout keeping in contact with the bottom and the body angle varying between 90° and 45° with the substratum. The swimming of both stopped, and the male moved its pelvic region toward the female. Copulation was closely observed by the second male of the squad and by another female, and physical contact with the mating couple was recorded, removing them from their initial position in the water column and both coming to lay their bodies directly over the substrate. Although they suffered this interference, the second male did not bite the female, nor was there any attempt to insert its clasper. Upon completion of copulation, the male and female did not immediately separate. The clasper remained inserted in the cloaca while both began to swim in opposite directions, forcing the withdrawal of the clasper and the couple’s separation.

The monitoring of the behavior of the specimens of *T. obesus* in the oceanic tank also allowed observation of the female’s escape strategies from unaccepted copulations. Several events were observed in the ocean tank, two of which were recorded on video and are described here (Video S2). The beginning of courtship followed the expected course and started with the chase by the male. The first strategy was to escape by fast swimming, avoiding the direct approach to the exposed pectoral fin and the necessary bite to anchor. If anchoring was successful, the female moved erratically and swam at different speeds while the male stopped swimming. Finally, the female made use of the substrate’s complexity to release the male’s bite, initiating a quick escape soon afterwards before the male could restart its approach.

### 3.2. Internal Morphology

**Figure 1:**
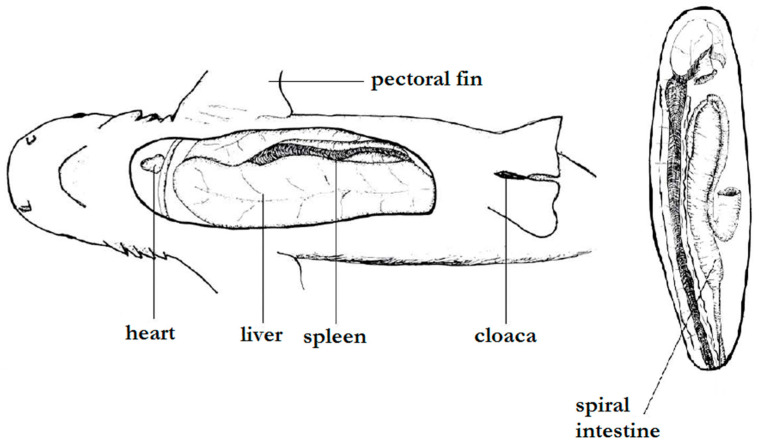
Basic internal anatomy of a carcharhinid shark (*Carcharhinus melanopterus*), showing the location of principal organs. Adapted from Crow and Brock [32].

**Figure 2:**
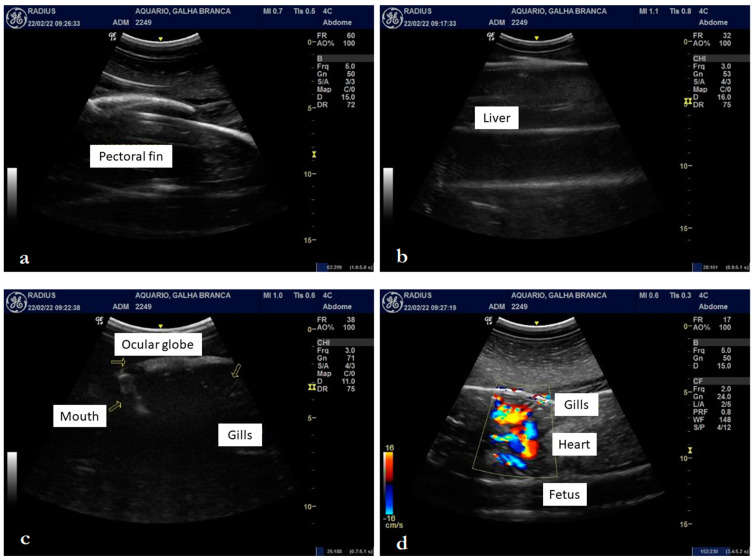
Ultrasonography of a female *Triaenodon obesus* (Rüppell, 1837) in late pregnancy: fetal orientation (brain/tail) and pectoral fins (**a**), hypoechogenic liver (**b**), eyes and gills (**c**), and heart with individualized chambers (**d**).

**Figure 3:**
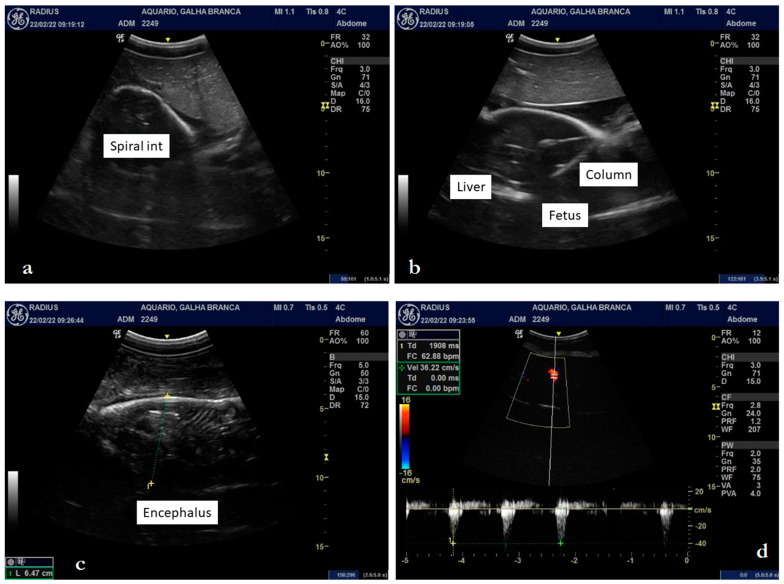
Ultrasonography of a female *Triaenodon obesus* (Rüppell, 1837) in late pregnancy: intestinal spiral and parietal stratification (**a**), cartilaginous skeleton, forming a posterior acoustic shadow artifact (**b**), biparietal diameter of 6.47 cm (**c**), and cardiac blood flow of 62 beats per minute (**d**).

Upon ultrasound examination, it was possible to detail a fetus with a well-defined brain, tail, and pectoral and dorsal fins, with well-defined gills and coelomic cavity (Figure 1, Figure 2a–d, and Figure 3a–d). The fetal liver was characterized by a hypoechoic, homogeneous parenchyma with regular contours, also showing the gallbladder with anechogenic content (Figure 2b). The kidneys were partially defined and presented a slightly heterogeneous hypoechogenic parenchyma. The intestinal spiral with the characteristic spiral pattern was evident, as was a defined parietal stratification (Figure 3a). The cartilaginous skeleton was hyperechoic and formed a moderate posterior acoustic shadow artifact (Figure 3b). It was possible to detail the cranial conformation, define the orbital fossa housing the anechoic eyeball with a hyperechoic capsule (Figure 2c), and evaluate the biparietal diameter, which measured approximately 6.47 cm (Figure 3c).

The vertebrae of the spine presented themselves as hyperechoic, individualized structures, homogeneously spaced, with an anechoic central area, possibly the spinal canal (Figure 3b). In the cross-section, the spinous processes of the vertebrae were also detailed. The gills were defined as parallel slits of alternating echogenicity (hyperechogenic and anechogenic) (Figure 2c), which, on color Doppler mapping, showed an evident signal, indicating the presence of fluid passage. The heart presented individualized chambers and an evident blood flow signal when we used the color Doppler tool (Figure 2d). With spectral Doppler, it was possible to measure the heart rate, which presented a value of 62 beats per minute at the time of the examination (Figure 3d). Considering that the average gestation period observed by Schaller [24] is 387 days and knowing that the neonate described above was born 12 days after the ultrasound examination, we calculated that it was approximately 375 days old. Therefore, the description of organogenesis by this method characterized the final third of the pregnancy.

### 3.3. Birth and Feeding

A *T. obesus* female of 145.0 cm TL and 20.1 kg TW (total weight) successfully completed her reproductive process with the birth on 7 March 2022 of a male of 58.0 cm TL and 1.15 kg TW. A second female of 136.4 cm TL and 19.0 kg TW gave birth to a female neonate of 56.4 cm TL and 1.0 kg TW on 7 May 2022. This was the first occurrence of the reproduction of a whitetip reef shark in an aquarium in Brazil. Both offspring were active, displaying constant swimming even though they rejected food for the first three and seven days, respectively. The first neonate fed for the first time on the third day, consuming 15 g of tilapia (*Oreochromis* sp.). The diet in the first trimester consisted of Brazilian sardine *Sardinella brasiliensis*, tilapia, manjubas *Anchoa* spp., shrimp *Penaeus* spp., and squid *Doryteuthis* spp. Over the initial three months, teleosts composed 76% of the diet, followed by shrimp (21%) and squid (3%). As for the second neonate born in 2022, feeding only started after seven days, and any food offered before then was rejected. The items offered in the diet were the same as those of the male, with tilapia accepted first, while in the following feedings, the male consumed mostly manjubas and sardines. The pregnant female isolated during the quarantine showed a marked reduction in her feeding during gestation, fasting for up to ten days, with erratic intervals in the following days until giving birth. Following veterinary recommendation and internal postpartum protocol, the recovering female was medicated via intramuscular injection with Ceftazidime 30 mg/kg and sodium methylprednisolone succinate 2 mg/kg, every 72 h, totaling five applications, due to a slight increase of leukocytes. Afterwards, she returned to her normal appetite and was transferred to the ocean tank.

## 4. Discussion

### 4.1. Courtship, Copulation, and Pregnancy

These records, made between 2021 and 2022, allowed us to evaluate the process of courtship with copulation and the gestation of the whitetip reef shark in an ex situ environment. The observations were compared with events already described in the literature and coincided with the copulations recorded previously. Whitney et al. [20] described the estimated period in the natural environment for the mating season and birth, based on photo identification made off the coast of Kona, Hawaii (USA). The authors pointed out a possible overlap of the birth period with the mating period, estimating that gestation spans one year. In the case reported here, the records of mating scars were made in June 2021, while births occurred between March and May, close to but shorter than the period reported for the species by Whitney et al. [20]. Comparing the mating record obtained in 2021 and the date of the first birth in 2022, the gestation period for the female born ex situ would have been 278 days, or just over 9 months. However, if the filmed copulation and the birth of the second neonate are considered, the gestation period would have been 339 days, which differs little from that observed for the species. Schaller [24] reported 5 births of *T. obesus* at the Steinhart Aquarium (San Francisco, CA, USA) between 2001 and 2004 and observed a longer gestation period, between 355 and 422 days, based on the first mating scar and the date of birth. Based on specimens captured off Indonesia by a commercial fishing fleet, White [33] observed two pregnant females of sizes close to our females (158.1 and 140.6 cm TL), with four formed embryos gestated by the first and three embryos found in the second, and estimated the total gestation time to be greater than six months.

Whitney et al. [34] observed three courtship events in the Coco Islands (Costa Rica), two of which involved only one male and one female. The beginning was recorded at mid-water, with the descent to the substrate provoked after the bite on the female’s pectoral fin and, consequently, the inhibition of swimming. The courtship concluded with copulation in only one of the events. The courtship attempts we observed in our oceanic tank were all initiated in mid-water. Similar to the observations made by Whitney et al. [34], most attempts did not result in copulation. Incomplete copulation occurred owing to the female’s detachment from the pectoral fin bite and the male and female’s inability to keep swimming in parallel and in close enough contact to allow a second bite to restart the descent to the bottom. Despite being in an ex situ environment, the female’s nonacceptance and escape strategies observed at AquaRio confirmed previous observations made in a natural environment [34] that indicated the success of copulation being largely dependent on acceptance from the female. Pratt Jr. and Carrier [35], in a review of reproductive behavior in elasmobranchs, noted that biting is apparently a universal characteristic of the group, although for larger species some degree of cooperation or acceptance by the female is necessary for actual copulation to occur. Copulation events observed or recorded within other elasmobranch species from our aquarium (*Ginglymostoma cirratum* and *Stegostoma tigrinum*) support this finding, especially for an event involving a female of *S. tigrinum*. In this observed event, after a tail bite, the female moved to the substrate and exposed its belly, even though the male did not conclude the copulation. In the case of *T. obesus*, the nonacceptance of courtship was associated with accelerated swimming, even after the pectoral bite, and erratic movement, which made it difficult for the male to chase. The cloaca was protected by the female’s body movement and the structural complexity of the habitat was used to enact the escape or disarm the bite.

Tricas and Le Feuvre [36] described two mating events for the species from observations on Molokini Island, Hawaii (USA), in a shallow (7 m) protected reef area. The description began with the male anchored to the female by biting the pectoral fin, the male’s right clasper inserted into the cloaca, and the bodies positioned with the head downward and the rest of the body suspended in the water column at an angle of 45°. The two copulation events observed in our oceanic tank coincided with the events described by Tricas and Le Feuvre [36], occurring shortly after the spiral descent. Whitney et al. [34] expanded on the findings of Tricas and Le Feuvre [36], having observed a larger set of courtships, copulations, and the functioning of the siphon bags. Whitney et al. [34] reported the presence and behavior of other males that did not participate directly in copulation but remained present and sometimes participated in the initial moment of copulation, biting the female’s pectoral fin to provoke the descent to the bottom, but separating soon after without copulating. In the two courtships with copulation recorded by us, the second male was observed circling the main couple, not having participated with any bite on the pectoral fin. In the first event, in 2021, the male’s physical contact caused the couple to leave their suspended and inclined position in the water column, but there was no insertion of the clasper by the second male, who moved away from the couple shortly afterwards. The most recent event did not present any interference from the second male other than constantly circling the couple during mating.

Studies on changes in behavior and feeding during pregnancy and in the period before birth are scarce in the literature for most species [37,38]. The whitetip reef shark is characterized by greater activity at night, remaining at rest for most of the day [39,40]. After monitoring the activity of adults by recording swimming acceleration and the intensity of breathing in neonates, Whitney et al. [39] pointed out that the latter assumed the pattern observed for adults soon after birth. The circumstances of the two neonates born in quarantine and in the ocean tank agreed with the observation made by Whitney et al. [39], with the same circadian pattern as adults being verified in neonates. Even so, the female born in quarantine and observed in more detail demonstrated more active diurnal swimming in the first day after birth before assuming the nocturnal pattern similar to that described for the species in the following days.

The feeding during the pregnancy of the first female of *T. obesus* was intermittent, being generally characterized by the acceptance of food every two days. It is important to note that the transfer process from the oceanic tank to the isolation in the quarantine tank was followed by a period of ten days in which the pregnant female remained within the expected activity pattern for an adult, but without accepting any food. Although we cannot assume this to be a feeding pattern of a pregnant whitetip reef shark, the disturbance of the transfer may have provoked enough stress to cause an interruption in the feeding of a pregnant female, despite it being a routine activity that does not cause the same reaction in nonpregnant females.

Given that it was the first pregnancy for the two females in question, in addition to the known relative sizes of the pregnant female and the embryo in *T. obesus*, the occurrence of only one offspring each could have been caused by the restricted space for their development. However, based on a visual census conducted in 21 reefs of the Great Barrier Reef (Australia) and records for 76 females, Robbins et al. [41] and Robbins [1] identified pregnancies of 1 to 4 offspring with an average of 2.07 neonates and showed no significant correlation with the size of the pregnant female or her age, with this being considered a particularly low level of fecundity compared to other species of Carcharhinidae.

### 4.2. Internal Morphology

Many of the studies devoted to the embryonic development of sharks have been carried out using oviparous species, given the ease of obtaining fertilized eggs to monitor the different stages of development [42]. In viviparous or ovoviviparous species, monitoring is more complex owing to restrictions on obtaining pregnant females found in the natural environment [43,44,45,46]. Research heavily relies on accidental captures by fishing of pregnant females at different stages of embryonic development to compose a sequence of embryonic stages [38,39]. Pregnancy monitoring in an ex situ environment offers the opportunity to monitor the same females from mating to birth, recording behavioral and physiological changes throughout the reproductive cycle [24]. The monitoring, however, depends on routine exams that allow the identification of pregnant females at the beginning of embryonic development. Maintenance of the usual behavioral pattern and the minor morphological alteration of the female during the initial and intermediate stages of gestation did not call attention to our whitetip reef shark until the last months before birth. While ultrasound exams are dedicated to the diagnosis and treatment of individuals suspected to be ill, monitoring by routine scheduled exams is necessary for viviparous species of elasmobranchs to enable the identification of pregnant females and recording of the early stages of embryo development.

Sharks of the Triakidae (*Mustelus* spp.) and Carcharhinidae (*Rhizoprionodon* spp.), placentotrophic species with annual reproduction, have concomitant pre-ovulatory vitellogenic follicles and well-developed embryos [45,46]. Although the ultrasound examination did not allow the visualization of follicles for the female at the end of gestation, a new courtship and attempt at copulation by the males started a few days after birth and the female’s return to the ocean tank. Considering that the average gestation period observed by Schaller [24] is 387 days and that the first neonate described here was born 12 days after the ultrasound examination, we assumed that the female was pregnant for approximately 1 year. Therefore, the description of organogenesis via this method characterized the final third of pregnancy. This study offers the first images of the development of a healthy embryo in the late gestation period of *T. obesus*.

Viviparous placental development has been recorded only in Selachii of the order Carcharhiniformes, in the families Leptochariidae, Triakidae, Hemigaleidae, and Carcharhinidae [22,47]. The literature describing the developmental stages for placental species is scarcer than that available for oviparous lecitotrophic species [42] or viviparous aplacental species with oophagia, histotrophy, and adelphophagy [48,49]. Although the stages for *T. obesus* have not yet been described, the whitetip reef shark embryo described herein, based on an ultrasound taken 12 days before birth, showed full structural development, with developed fins and skeleton, as well as internal organs without apparent anomalies. Behavior, feeding patterns, and routine veterinary examinations in the months following birth confirmed that both newborns were fully developed, healthy individuals.

### 4.3. Birth and Feeding

The ex situ reproduction of a shark species classified as vulnerable, such as *T. obesus* [19], highlights the need to integrate public aquariums in the consolidation of a viable ex situ population, according to criteria already outlined by Buckley et al. [50], and an essential step for this is the reproductive success and genetic variability of this population. Schaller [24] described four litters of *T. obesus* obtained between 2001 and 2003 in work conducted at the Steinhart Aquarium (USA), providing precise data on courtship, pregnancy, and parturition for the species. Portnoy et al. [51] described the birth of a stillborn whitetip reef shark at the BioPark Aquarium (USA), with no apparent reason for death or signs of trauma or malformation. It is important to note that Portnoy et al. [51] also pointed out that all parthenogenesis cases reported for sharks generated females, which initially suggested that the female pup born at AquaRio could have been a parthenogenetic event. Still, photographic records and recordings obtained in 2021 made it possible to follow courtship and copulation events among the *T. obesus* group, supporting the suspicion that the births were the product of sexual reproduction. The appearance of bite scars in June 2021 and the presence of a male pup also suggested that parthenogenesis did not take place. Furthermore, a DNA analysis is going to be conducted to establish if the same male was responsible for both shark pups. The diet of adult whitetip reef sharks consists mainly of teleosts (Holocentridae, Lutjanidae, Pomacentridae, Scaridae, Acanthuridae, Balistidae, Mullidae), which can comprise 90% of their food, although mollusks and crustaceans have also been reported [29,52]. The food preference displayed by the neonates reflected the described diet for adults, consisting mainly of teleosts, but with a relevant inclusion of penaeid shrimps.

## 5. Conclusions

The study compared the data available for the reproduction of *T. obesus* in the literature, based mostly on in situ observations, and the case reported by Schaller [24] for births registered at the Steinhart Aquarium (USA), making it possible to corroborate the courtship stages already reported, as well as to identify female escape strategies and contribute data on gestation duration and the initial development of neonates and their food preference. Furthermore, ultrasonography is a noninvasive examination technique and it allowed us to detail fetal organogenesis and study the viability of the fetus in whitetip reef sharks, with this being the first ultrasonographic description of fetal organogenesis in the species and the first report of reproduction of the species in a Brazilian aquarium.

Completing the reproductive cycle of endangered elasmobranchs in an ex situ environment is another relevant step that may lead to an effective plan for the preservation of these species. While natural populations are under pressure from habitat loss and overfishing, a healthy population kept safe from these impacts will enable a species reintroduction plan to be implemented after minimum living conditions are restored for the species in their original distribution. This strategy is even more vital when it comes to coral reefs, highly productive ecosystems with rich fauna and complex food webs. Existing literature reports several threats already affecting these communities, within which *T. obesus* is an essential predator. Reproductive success will offer hope for Brazilian species of Carcharhinidae, as many share characteristics with the whitetip reef shark, such as being viviparous placentotrophs. The lessons learned from whitetip reef sharks under human care may assist in the development of protocols that help with the proper handling of other carcharinid sharks in ex situ conditions, with the possibility of increasing the chances of successful reproduction of endangered species, such as the highly threatened sandbar shark *Carcharhinus plumbeus* (Nardo, 1827) and the small-tail shark *Carcharhinus porosus* (Ranzani, 1839).

## Data Availability

Not applicable.

## Supplementary Materials

The following supporting information can be downloaded at: https://www.mdpi.com/article/10.3390/ani12233291/s1, Video S1: Courtship and copulation of *Triaenodon obesus* (Rüppell, 1837), recorded at the Rio de Janeiro Marine Aquarium. Video S2: Failed attempt at courtship of *Triaenodon obesus* (Rüppell, 1837), recorded at the Rio de Janeiro Marine Aquarium.
